# Two-millimetre diameter operative arthroscopy of the ankle is safe and effective

**DOI:** 10.1007/s00167-020-05889-7

**Published:** 2020-02-17

**Authors:** Tobias Stornebrink, J. Nienke Altink, Daniel Appelt, Coen A. Wijdicks, Sjoerd A. S. Stufkens, Gino M. M. J. Kerkhoffs

**Affiliations:** 1 Department of Orthopedic Surgery, Amsterdam Movement Sciences, Amsterdam UMC, Location AMC, University of Amsterdam, Meibergdreef 9, 1105 AZ Amsterdam, The Netherlands; 2 Academic Center for Evidence-based Sports medicine (ACES), Amsterdam UMC, Amsterdam, The Netherlands; 3 Amsterdam Collaboration for Health and Safety in Sports (ACHSS), International Olympic Committee (IOC) Research Center, Amsterdam UMC, Amsterdam, The Netherlands; 4 Department of Orthopedic Research, Arthrex GmbH, Munich, Germany

**Keywords:** Arthroscopy, NanoScope, Ankle, Innovation, Safety, Efficacy

## Abstract

**Purpose:**

Technical innovation now offers the possibility of 2-mm diameter operative arthroscopy: an alternative to conventional arthroscopy that no longer uses inner rod-lenses. The purpose of this study was to assess whether all significant structures in the ankle could be visualized and surgically reached during 2-mm diameter operative arthroscopy, without inflicting iatrogenic damage.

**Methods:**

A novel, 2-mm diameter arthroscopic system was used to perform a protocolled arthroscopic procedure in 10 fresh-frozen, human donor ankles. Standard anteromedial and anterolateral portals were utilized. Visualization and reach with tailored arthroscopic instruments of a protocolled list of articular structures were recorded and documented. A line was etched on the most posterior border of the talar and tibial cartilage that was safely reachable. The specimens were dissected and distances between portal tracts and neurovascular structures were measured. The articular surfaces of talus and tibia were photographed and inspected for iatrogenic damage. The reachable area on the articular surface was calculated and analysed.

**Results:**

All significant structures were successfully visualized and reached in all specimens. The anteromedial portal was not in contact with neurovascular structures in any specimen. The anterolateral portal collided with a branch of the superficial peroneal nerve in one case but did not cause macroscopically apparent harm. On average, 96% and 85% of the talar and tibial surfaces was reachable respectively, without causing iatrogenic damage.

**Conclusion:**

2-mm diameter operative arthroscopy provides safe and effective visualization and surgical reach of the anterior ankle joint. It may hold the potential to make ankle arthroscopy less invasive and more accessible.

## Introduction

Arthroscopy is gaining importance in the treatment of ankle pathology [17]. Yet, it is still a relatively invasive procedure, with hyp(er)aesthesia of the superficial peroneal nerve and iatrogenic damage to articular cartilage as possible complications [16, 19]. Most procedures require spinal or general anaesthesia, which is associated with the risk of additional complications [9, 10] and the need for an operating theatre. All this causes the barrier to resort to a simple arthroscopic intervention to remain high.

A less invasive and more accessible way of arthroscopy may benefit ankle surgery in both diagnosis and in treatment. Alternatives to conventional rod-lens systems were first developed in the 1990s and seemed to support such an arthroscopic technique. However, these, as they were called, needle arthroscopes yielded images of inferior quality and their diagnostic accuracy was accordingly limited [20]. The inferior image quality, in combination with a lack of tailored surgical tools, restricted interventional possibilities. In addition, the systems consisted of large and cumbersome machinery [2]. Not surprisingly, traditional needle arthroscopy was not added to the arsenal of the orthopaedic surgeon.

Recently, a new alternative to rod-lens arthroscopy was released that uses a disposable, chip-on-tip arthroscope [15]. This novel technology promises to facilitate arthroscopy with a semi-rigid, durable combination of arthroscope and cannula, its total diameter just over two millimetres. Compared to the needle arthroscopes of the past, this novel technique asserts to benefit from substantially improved image quality and general practicality. Whilst image quality increased, the size of the scope and supporting devices decreased. In addition, the system comes with arthroscopic surgical instruments that are specifically tailored to 2-mm diameter arthroscopy. These advancements seem to introduce the possibility of 2-mm diameter operative arthroscopy, which might dramatically increase the accessibility of arthroscopic ankle interventions. Nonetheless, the true safety and efficacy of 2-mm diameter operative arthroscopy of the ankle yet remains unknown.

With new technology, new scientific evidence is required. Therefore, the aim of this study was to evaluate whether the newly introduced 2-mm diameter operative arthroscope could effectively visualize and surgically reach all relevant structures in the anterior ankle joint, without inflicting iatrogenic damage.

## Materials and methods

### Specimens

Ten non-paired, fresh-frozen, human lower leg specimens without a known or apparent history of ankle surgery were obtained through the Science Care donation programme (4 male, 6 female, mean age 72 years). Specimens were de-identified and, therefore, institutional review board approval was not required. All leg specimens were amputated at the level of the proximal tibia for fixation purposes.

### Arthroscope

A novel arthroscopic system (NanoScope™, Arthrex, Naples, FL, USA) was used. This system consists of a disposable handpiece and a tablet-like, medical-grade control unit. A LED light source, an illumination system and a detection system are all included in the handpiece. The handpiece tube is 9.5-cm long, semi-rigid and has a 1.9-mm outer diameter. It carries the detection system at its distal end (chip-on-tip technology). The scope’s direction of view is 0°, with a 120° field of view. The sensor chip has a pixel number of 400 × 400. The system comes with tailored accessories such as cannulas and arthroscopic instruments for interventional purposes. The cannula has a 2.26-mm outer diameter. It was used for scope insertion and to maintain joint access. The cannula can be connected to distention systems and distention media pass between the scope and inner cannula wall.

### Procedure

**Fig. 1:**
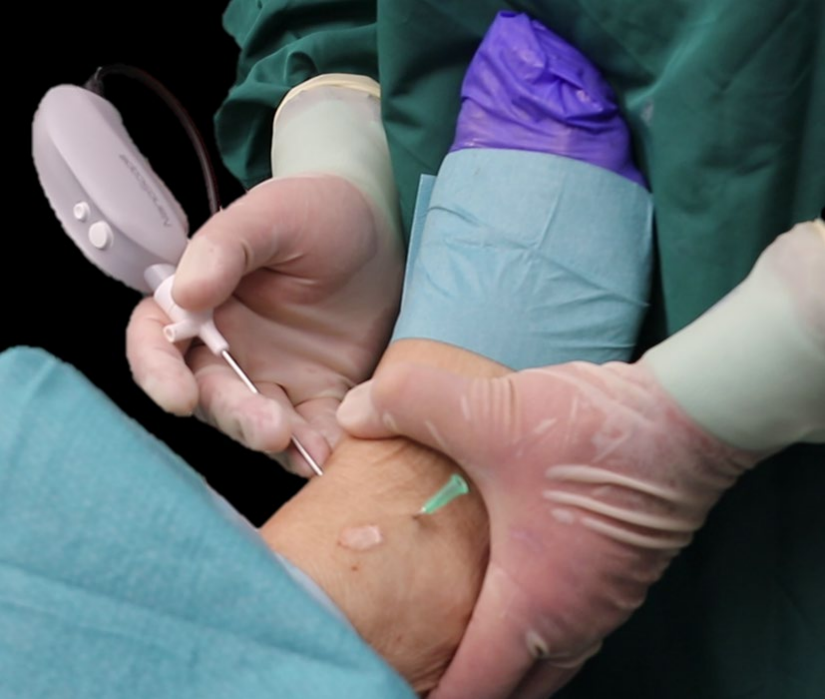
Outside view during a 2-mm diameter arthroscopic procedure. The arthroscope is introduced through the anteromedial portal. A green needle marks the anterolateral portal

An experienced, sports medicine and ankle fellowship-trained orthopaedic surgeon (GK) performed a protocolled procedure in each specimen. The specimens were rigidly fixated to a workbench, with the ankle in supine position and tested for ligamentous laxity and range of motion. In each specimen, an anteromedial, anterolateral and anterocentral portal were created, all as described by Golano et al. [8]. To obtain portal access, 2-mm skin incisions were made parallel to the relevant surrounding anatomic structures, as recommended [8]. With the help of a 1.9-mm diameter blunt obturator, the 2.26-mm diameter cannula was inserted through the joint capsule at the anteromedial portal. The obturator was removed and the arthroscope was introduced through the cannula (Fig. 1). No distraction was applied to the ankle joints during the procedures. A laparoscopic insufflator (Arthrex, Naples, FL, USA) distended the joints with carbon dioxide.

Two ankle fellowship trained, experienced orthopaedic surgeons (GK, SAS) systematically recorded visualization of the anatomic entities that are listed in Table 1. The entities listed in this table were modified from Ferkel (1996) and reflect a complete set of structures that should be visualized for proper arthroscopic evaluation of the anterior ankle [5]. The anteromedial portal was used for visualization. It was recorded whether the visualization of each structure was successful, resulting in a dichotomous outcome measure. In addition, any occurring limits to visualization and any additional portals required for successful visualization were critically documented.

**Table 1:** Structures visualized during diagnostic evaluation of the anterior ankle joint

Structures	
1	Deltoid ligament
2	Medial gutter
3	Medial talus
4	Central talus
5	Lateral talus
6	Talofibular articulation (ATFL, AITFL)
7	Lateral gutter
8	Anterior gutter

**Fig. 2:**
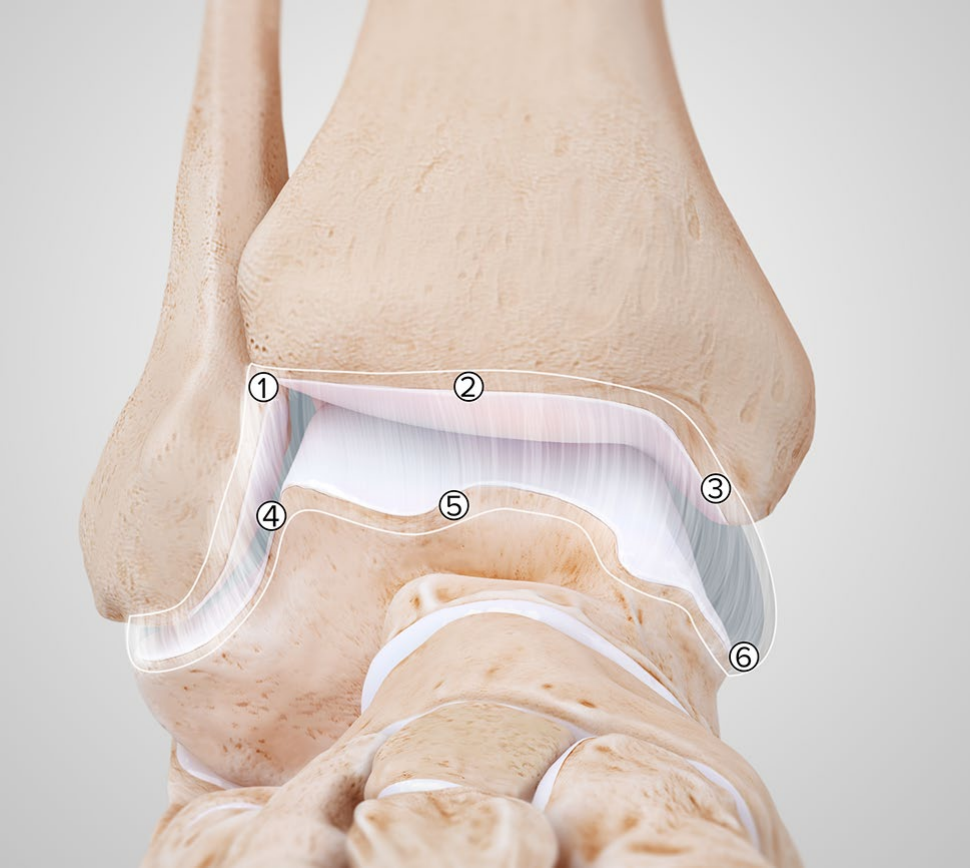
Points of biopsy on the anterior ankle capsule to determine surgical reach. The capsule as delimited by the white line. Cranial points 1 (most laterally), 2 (mid-tibial), 3 (most medially) and caudal points, 4 (most laterally), 5 (mid-talar), 6 (most medially)

Efficacy of surgical reach was determined in the anterior ankle joint space as well as on the articular surfaces of the talus and tibia. Six points on the anterior ankle capsule provided a proxy for reach in soft tissue (Fig. 2). A 2-mm diameter arthroscopic biter (NanoBiter™, Arthrex, Naples, FL, USA) was used to take biopsies of the joint capsule at each of these points. Surgical reach of each point was confirmed under visualization with the arthroscope, with pictures taken at the moment of biopsy for documentation. A 2-mm diameter retractable probe (NanoProbe™, Arthrex, Naples, FL, USA) was used to identify the most posterior part of the talar and tibial articular surfaces that were within safe surgical reach, i.e. reachable without inflicting damage to the cartilage or surrounding tissues. A line was etched in the cartilage at this most posterior border. During the interventional procedures, visualization was obtained from the anteromedial portal and surgical instruments were introduced through the anterolateral portal. No cannula was used for the instruments. Successful biopsies and etched lines, limits to surgical reach and any additional portals required for successful biopsy or etching were critically documented by a second investigator (TS).

At the end of the arthroscopic procedures, all portal tracts were replaced with 1.8-mm diameter Kirschner-wires and detailed anatomic dissection was performed. A calibrated dial calliper with an accuracy of 0.01 mm was used to measure the distance between portal tracts and surrounding anatomic structures. Measurements were rounded to the nearest decimal number of millimetres. Tables 2, 3 and 4 list the dissection protocols for each portal, all based on Golano et al. [8]. Finally, the ankles were disarticulated at the level of the talocrural joint, and the articular surfaces of talus and tibia were perpendicularly photographed and visually inspected by the two ankle-specialized orthopaedic surgeons for signs of macroscopically apparent iatrogenic damage to the cartilage.

**Table 2:** The anteromedial portal tract and its surrounding structures

Anatomic entity	Shapiro–Wilk W (*p* value)	Mean distance	95% confidence interval	Median distance	Interquartile range	Cases of contact
Great saphenous vein and nerve	0.91 (0.296)	6.8	4.2–9.4	–	–	0
Anterior neurovascular bundle	0.88 (0.141)	14.3	10.6–18.0	–	–	0

**Table 3:** The anterolateral portal tract and its surrounding structures

Anatomic entity	Shapiro–Wilk W (*p* value)	Mean distance	95% confidence interval	Median distance	Interquartile range	Cases of contact
Intermediate dorsal cutaneous nerve	0.80 (0.015)	–	–	2.2	1.4–3.3	1
Anterior neurovascular bundle	0.86 (0.067)	8.8	4.8–12.9	–	–	0

**Table 4:** The anterocentral portal tract and its surrounding structures

Anatomic entity	Shapiro–Wilk W (*p* value)	Mean distance	95% confidence interval	Median distance	Interquartile range	Cases of contact
Medial dorsal cutaneous nerve	0.90 (0.229)	5.2	2.0–8.5	–	–	1
Anterior neurovascular bundle	0.78 (0.007)	–	–	0.0	0.0–1.6	6

### Analysis

**Fig. 3:**
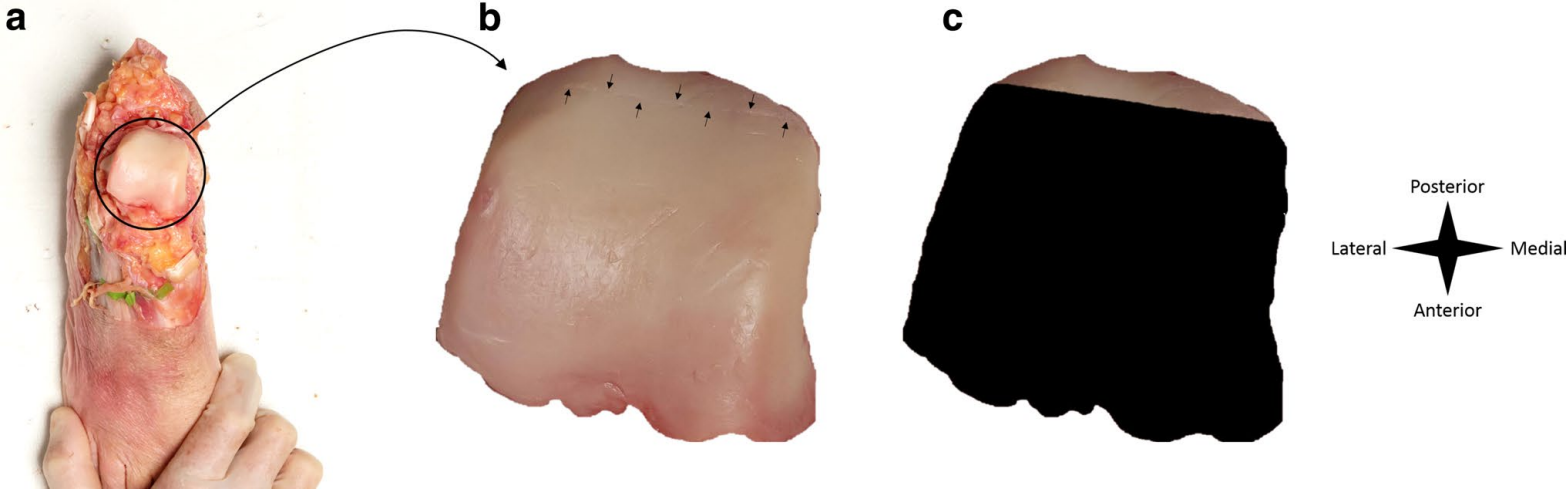
Analysis of surgical reach on a talar specimen. **a**: Disarticulated ankle. **b**: Talus as analysed, with the etched line between arrows. **c**: Surgically reached area in black

A Shapiro–Wilk test established the distribution of each set of distances between portal tracts and surrounding structures. In the case of normal distributions, mean distances and confidence intervals were reported. Otherwise, medians and interquartile ranges were presented. StataSE 15 was used for these analyses (StataCorp. 2017, College Station, Texas, USA). The pictures of the talar and tibial articular surfaces were uploaded into ImageJ graphic-analytic software (ImageJ, U.S. National Institutes of Health, Bethesda, Maryland, USA). In ImageJ, the line that was etched in the cartilage was traced. It was assumed that the articular surface anterior to this line was reached with the probe and that the surface posterior to this line was not reached. In ImageJ, the surface area that was reached was then calculated as a percentage of the entire articular surface area of either talus or tibia (see Fig. 3 for an example of the process in ImageJ). Percentages were rounded to the nearest integer. Finally, the average percentage surface area of the talar dome and the tibial plafond that could be reached was derived for the aggregate of specimens.

## Results

**Fig. 4:**
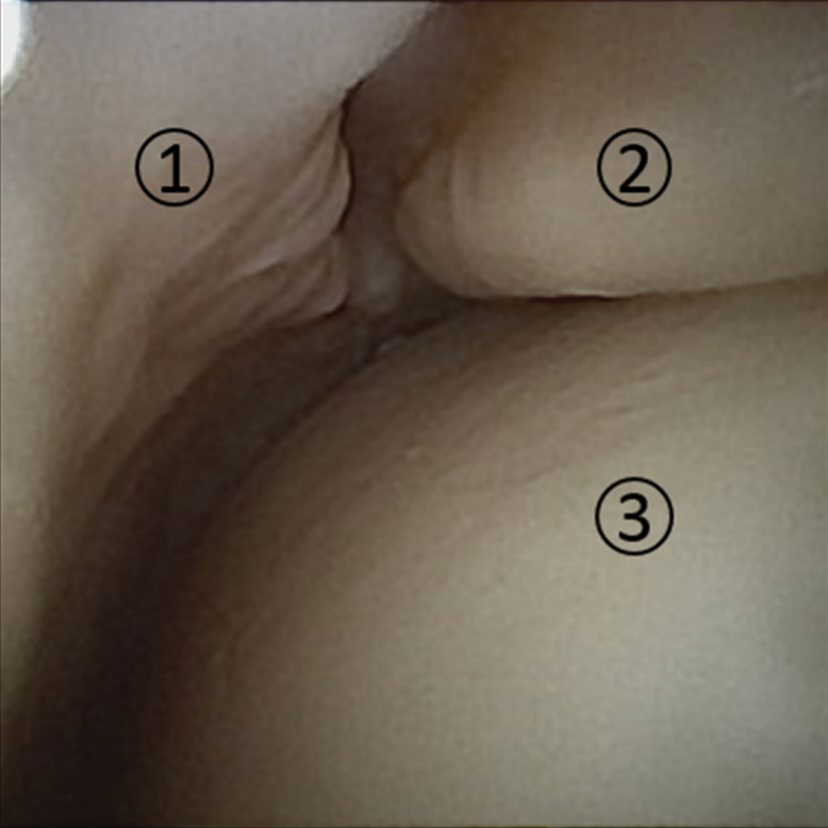
Visualization of the ankle joint during 2-mm diameter arthroscopy. 1: Joint capsule, 2: tibia, 3: talus

There was no evidence for ligamentous laxity in any specimen upon physical examination. Passive range of motion was unrestricted in all ankles, with the exception of two specimens where plantar flexion was decreased to < 15°. It was possible to visualize all the structures listed in Table 1, in every specimen (see Fig. 4 for an example of the intra-articular view). Besides limited degenerative changes, there were no signs of pre-existing intra-articular pathology. None of the specimens needed a portal additional to the anteromedial portal for proper visualization. In the two ankles with plantar flexion decreased to < 15°, the view of the posterior talar and tibial surface was harder to obtain. However, switching the scope to the anterolateral and anterocentral portals did not improve visualization.

**Fig. 5:**
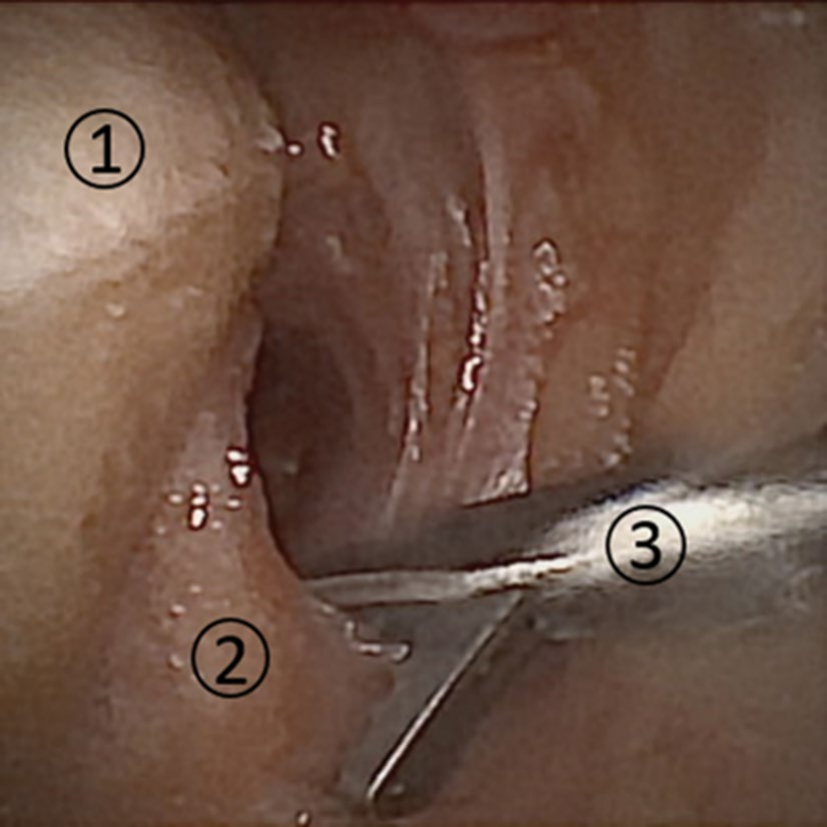
Biopsy of the ankle capsule with arthroscopic biter, tailored to 2-mm diameter, operative arthroscopy. 1: Talus, 2: joint capsule, 3: arthroscopic biter

Biopsies of the joint capsule were successfully obtained at the six proxy points in every specimen (see Fig. 5 for an example). Likewise, a borderline was successfully etched on the cartilage of the articular surface of the talus and tibia in every specimen. In none of the specimens a portal in addition to the anterolateral portal was required for either biopsies or etching.

In none of the specimens did the anteromedial portal tract collide with neurovascular structures. The anterolateral portal tract was in contact with the intermediate cutaneous branch of the superficial peroneal nerve in one case, though it caused no macroscopically apparent damage. The anterocentral portal tract was in contact with the medial cutaneous branch of the superficial peroneal nerve in one case, and with the anterior neurovascular bundle in six cases. In specimens with a more laterally directed anteromedial portal or a more medially directed anterolateral portal, the distance to the anterior neurovascular bundle was substantially under average. All distances between portal tracts and surrounding tissue were normally distributed, except for the distances between the anterolateral portal and the intermediate cutaneous branch of the superficial peroneal nerve and the distances between the anterocentral portal and the anterior neurovascular bundle. Distances, and their normality tests, are listed in Tables 2, 3 and 4 for each portal.

The analysis in ImageJ showed that for the aggregate of specimens, an average of 96% (SD 4%) of the talar, and 85% (SD 9%) of the tibial articular surface was reached with the probe. Reach was consistently compromised at the most posterior parts of the talar dome and tibial plafond, at the transition towards the posterior gutter. Reach was below average in the two joints with decreased plantar flexion, with a coverage of 93% and 86% of the respective talar surfaces, and 82% and 91% of the respective tibial surfaces. Visual inspection of the disarticulated talar dome and tibial plafond did not show iatrogenic damage to the cartilage.

## Discussion

The main finding of this study was that 2-mm diameter arthroscopy, a novel alternative to conventional rod-lens arthroscopy, provides effective visualization and operative reach of the anterior ankle, without inflicting iatrogenic damage when using the anteromedial and anterolateral portals. The entire anterior joint was visualized with the use of only one, anteromedial portal. With the addition of second, anterolateral portal, tailored surgical instruments successfully took biopsies of the entire anterior joint capsule and reached 96% of the talar dome, and 85% of the tibial plafond on average. No iatrogenic damage was inflicted during portal placement or arthroscopic procedures.

All beforehand selected anatomic structures were easily identified. This highlights the substantial increase in image quality of the 2-mm diameter arthroscopic system that was used in this study, compared to previous alternatives to rod-lens arthroscopes as for example used in the knee [11]. Only one portal was needed for visualization of the ankle joint. This anteromedial portal maintained a relatively high mean distance to neurovascular structures, and in no case made contact to such critical tissue. The anteromedial portal is indeed often regarded as safe, also in conventional arthroscopy [4, 14]. The need for potentially only one, safe portal for arthroscopic visualization supports the minimally invasive nature of 2-mm diameter arthroscopy and might lower the barrier to its use. It is important to note that it was harder to obtain visualization posteriorly in two specimens hampered by insufficient plantar flexion. Whilst the anterocentral portal is often found to provide a wider view [8], it provided no better images in these two cases. Given its additional convincing risk of harm to in particular the anterior neurovascular bundle, use of the anterocentral portal should be avoided. Ultimately, the suitability of 2-mm diameter arthroscopy in an ankle with decreased mobility might depend on the indication.

An anterolateral portal was added to introduce arthroscopic surgical instruments, specifically designed for 2-mm diameter operative arthroscopy. With the anterolateral portal, a wide surgical reach on soft tissue and articular cartilage was obtained. The portal was in contact with the intermediate cutaneous branch of the superficial peroneal nerve in one specimen but caused no macroscopically apparent damage to the nerve. Nonetheless, temporary post-operative hypaesthesia or paraesthesia in the nerve’s distribution area might be expected to occasionally occur after 2-mm diameter arthroscopic interventional procedures, as also commonly seen after conventional arthroscopy [4, 6]. Blunt trocar introduction, careful physical examination and transillumination with the arthroscope might help to avoid nerve contact [6, 8]. It should also be noted that the anterolateral portal tract gets close to the anterior neurovascular bundle when the portal is introduced in a more medial direction. It should hence be directed posteriorly during portal placement. For certain procedures, it might be feasible to use an accessory anteromedial portal instead of an anterolateral portal, which is of less risk to neurovascular structures [8].

A comparison between the results of the current study and previous reports on the safety of arthroscopy of the ankle shows substantial variability. For example, where this study found an average distance of 6.8-mm between the anteromedial portal and the great saphenous vein, previous studies reported distances of 5.4-mm [18], 9.0-mm [4], and 10.7-mm [13]. Whilst this and previous studies do provide a general idea of which portals can safely be used for arthroscopy, the variability in anatomic distributions underscores the need for careful introduction of arthroscopic portals. Whilst a recent clinical study reported that macroscopically visible iatrogenic cartilage lesions are inflicted in 31% of all arthroscopic ankle procedures [16], no such iatrogenic damage was inflicted in the current study. This may be a result of careful portal placement, but a potential risk-reducing effect of the small-diameter and semi-rigid frame of the novel arthroscope should also be considered, especially in the narrow ankle joint.

Apart from reports on semi-guided synovial biopsies in rheumatology with mainly older systems [1, 7, 12], previous alternatives to rod-lens arthroscopes were never evaluated for interventional use. The novel alternative system that was used in this study, however, showed a large and safe surgical reach, which might be attributable to the recent big leap forward in image quality. The cadaveric results of this study imply that with this novel system, 2-mm diameter arthroscopy can now be used for operative intervention in the ankle. It may be valuable to further explore the potential of for example visually guided injections and biopsies, loose body removal, and precise application of indicated biologics. It should even be possible to perform the simplest procedures as biopsies and injections along the arthroscope itself, with the arthroscope and instrument simultaneously advanced through the anteromedial portal. This would further increase safety and accessibility of procedures and tailored cannulas should be developed for this use in the future. The large reach on the talar dome and tibial plafond potentially opens the possibility for treatment of small chondral defects [3]. In addition, 2-mm diameter arthroscopy might be a valuable adjunct during conventional arthroscopy or open procedures.

This study should be considered in the light of its limitations. It studied 2-mm diameter arthroscopy of the ankle in a cadaveric setting only. In this environment, it was possible to visualize and reach all predetermined structures in the ankle. Subjectively, image quality was indeed remarkably good, although of lower quality compared to the newest 4 K conventional rod-lens arthroscopes. An objective assessment of potential differences in diagnostic accuracy between conventional and 2-mm diameter arthroscopy might be of benefit to future literature. In addition, visualization and surgical reach might be harder obtain in a patient setting, as for example blood and debris might render the procedure more challenging. In general, the clinical merit of 2-mm diameter arthroscopy of the ankle will have to be defined in future patient studies.

This study indicates that 2-mm diameter operative arthroscopy might be a valuable tool for the ankle surgeon. It might allow for arthroscopy in a minimally invasive manner, under local anaesthesia, either in the operating theatre or the outpatient office. Compared to current practice, this would allow for diagnostic, interventional and second-look procedures to be performed at substantially reduced risk, time and costs. The results of this study may be used in clinical practice to guide the implementation of 2-mm diameter operative arthroscopy of the ankle.

## Conclusion

In a cadaveric setting, 2-mm diameter operative arthroscopy provided excellent visualization and surgical reach of the anterior ankle joint, without inflicting iatrogenic damage.
